# Caplan’s Syndrome with a twist

**DOI:** 10.31579/2690-4861/007

**Published:** 2020-01-22

**Authors:** Janaki Deepak, Blaine Kenaa

**Affiliations:** Division of Pulmonary and Critical Care Medicine, Baltimore VA Medical Health Center, Baltimore, MD 21201, USA.

**Keywords:** caplan’s syndrome, rheumatoid arthritis, chronic silica inhalation, proteinuria, inorganic dust, peripheral

## Abstract

Caplan’s syndrome is seen in patients with rheumatoid arthritis (RA) and chronic silica inhalation. We present a patient with RA who presented with multiple pulmonary nodules. Biopsy of the nodules revealed silica crystals under polarized light. He continued treatment for RA and his pulmonary nodules remained stable. However, he subsequently developed renal failure with nephrotic range proteinuria. We discuss silica and the associated autoimmunity in patients with chronic occupational exposure.

**Background:**

Caplan’s syndrome also known as rheumatoid pneumoconiosis is a disease entity that is seen in patients with rheumatoid arthritis (RA) exposed to chronic silica and inorganic dust [1,2]. Classically, they form peripheral well-defined pulmonary nodules with characteristic silica retained in the necrobiotic center. In addition, epidemiological data has shown some association with silica and autoimmunity [3]. We present a case of silica and asbestosis exposure in a patient with rheumatoid arthritis who developed rheumatoid pneumoconiosis and subsequent renal failure. We highlight this rare disease, progression as well as other associated complications.

## Case Presentation

**Figure 1:**
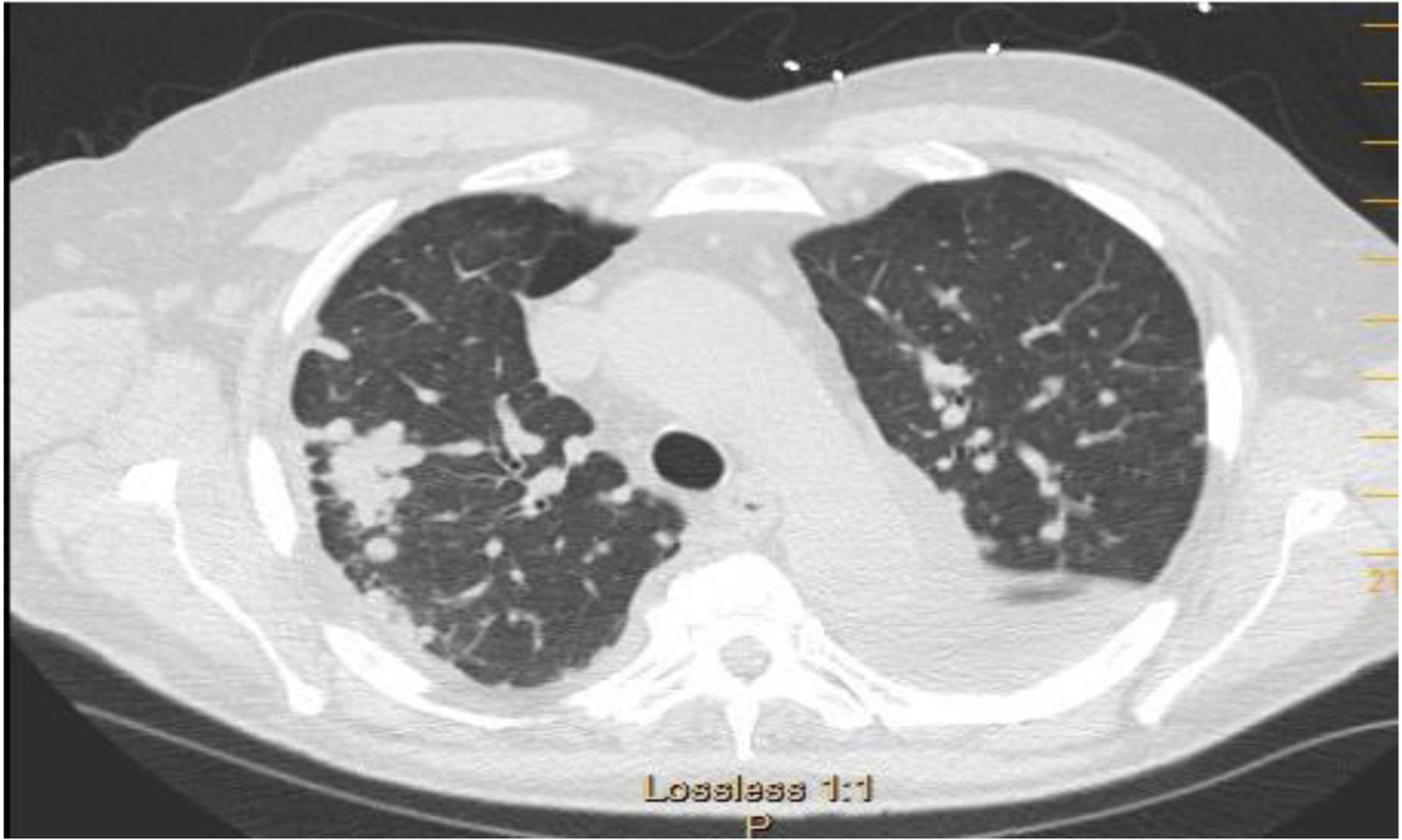
Axial CT chest showing nodules coalescing in to one large conglomerate. Black arrow pointing to coalescing nodules.

**Figure 2:**
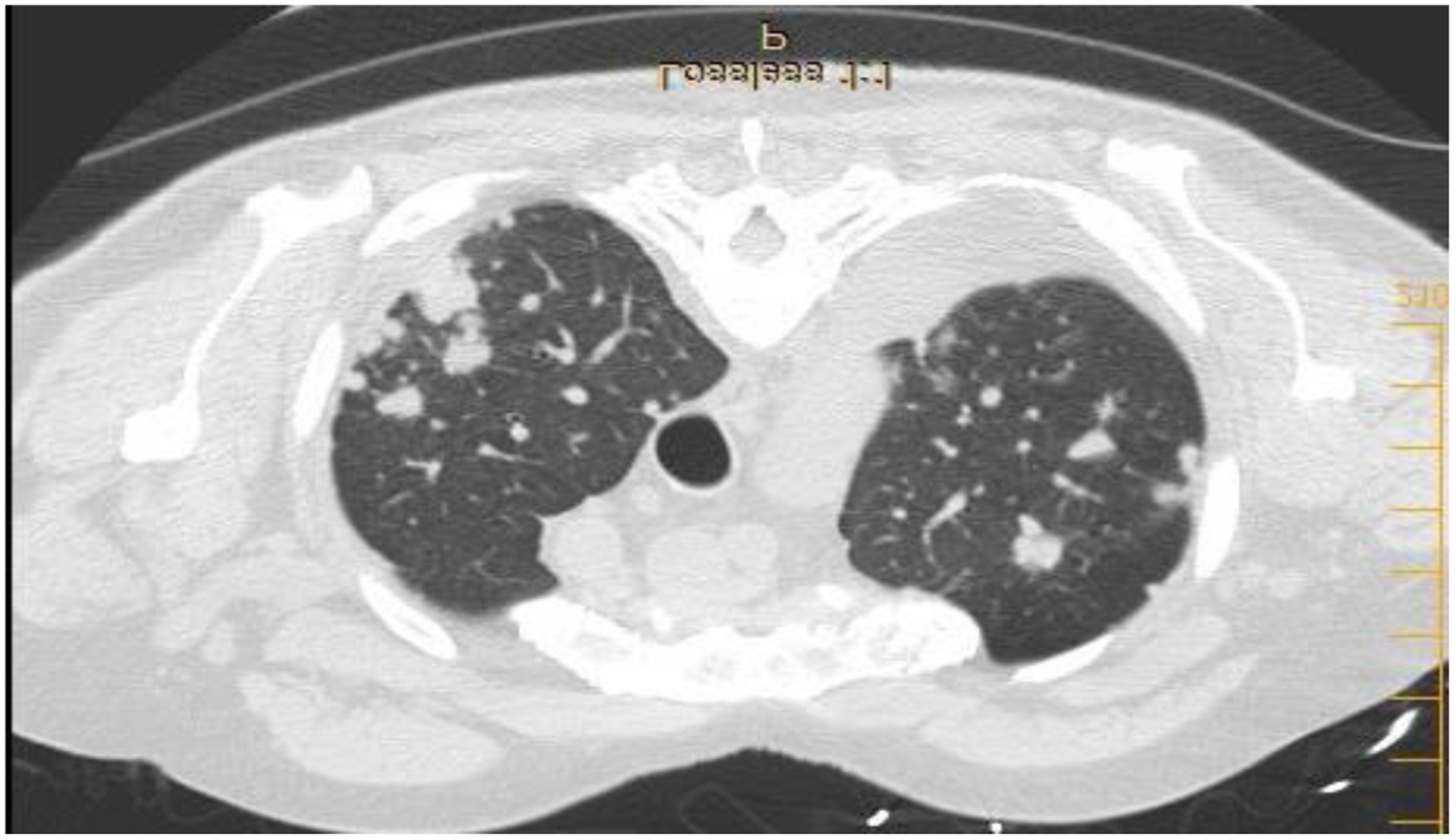
Axial CT chest showing multiple pulmonary nodules in the right upper lobe. Black arrow pointing to RUL nodules

A 73 y/o man with no smoking history and a diagnosis of RA was referred to the pulmonary clinic for multiple pulmonary nodules in a peri-lymphatic pattern (Figure 1). Patient was noted to have progressive pulmonary nodules that have been increasing in size (Figure 2).

His rheumatologist planned to start him on TNF-alpha inhibitors for his RA and wanted to evaluate for infections and malignant causes prior to proceeding with the treatment. He was asymptomatic and denied any respiratory complaints. At the time of presentation, he was being treated with leflunomide, hydroxychloroquine and sulfasalazine. Pt is a Navy veteran with exposure to asbestos while he was in the Navy. Following his discharge from the Navy, he worked 10 years overseeing production of plastic and metal parts for guided radar systems. He then transitioned to working in residential construction, hanging, siding and replacing windows. He also worked on restoring brick on old houses by stripping off paint for about 5 years.

**Figure 3:**
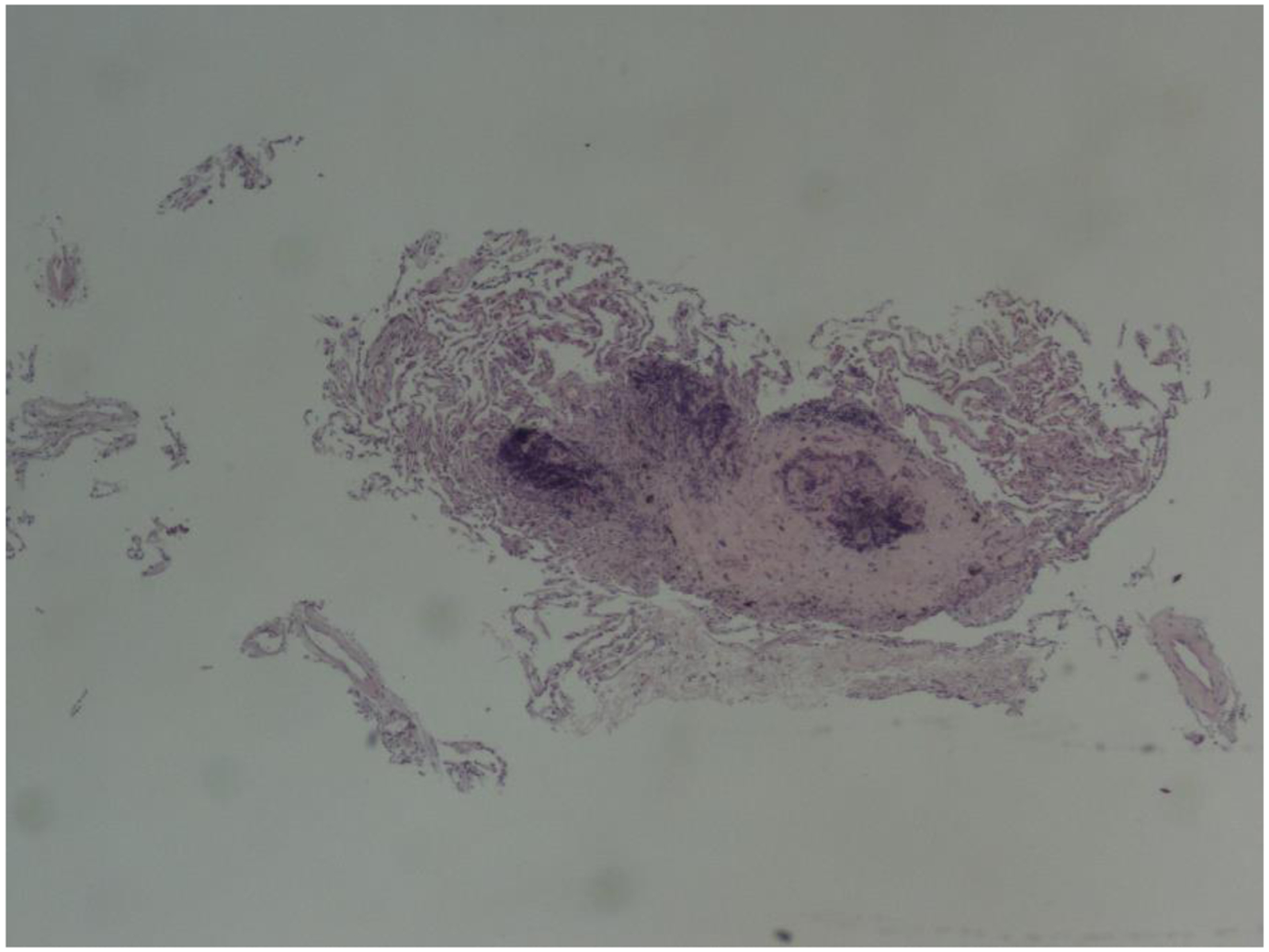
Pathology of the necrobiotic nodule from the transbronchial biopsy of the Right upper lobe nodule.

Pulmonary function test (PFT) revealed mild obstructive ventilator defect with FEV1/FVC 66%, FEV1 of 3.13L, 94% predicted. He underwent bronchoscopy with transbronchial biopsies of the right upper lobe nodules. Pathology revealed a necrotic tissue (Figure 3) with central silica crystals visualized under polarized light which was consistent with silico-anthracotic nodules. The biopsies were negative for malignancy. There was no evidence of infection on bronchoalveolar lavage. His AFB and fungal cultures were also negative.

Three years following his biopsy, the patient presented with lower extremity edema and dyspnea on exertion. He was found to have worsening renal failure with subsequent renal biopsy showing a mixture of minimal change disease (MCD) and focal segmental glomerulosclerosis (FSGS). The staining was negative for amyloid and no silica deposits were identified. He also underwent right heart catheterization that showed mild pulmonary hypertension with mean PAP of 33. CT chest did not show worsening or progression of the nodule

## Discussion

Since it was first described by Caplan in 1953, the exact correlation between RA, pneumoconiosis and autoimmunity has been a topic of debate [2]. Silica is recognized as a pathogen associated molecular pattern (PAMP) which stimulates macrophages and subsequent release of cytokines like TNF-alpha and IL-1 [1]. This process triggers a cyclical pattern of macrophage apoptosis and chronic inflammation generating fibroblast deposits and tissue destruction. The cytokines further stimulate the adaptive immune system with subsequent increased production of autoantibodies and immune complex [1].

Autoimmunity is not isolated to patients with RA, nor is it only seen in silica. Epidemiological data suggests that similar correlation exists between asbestos exposure and autoimmunity [3]. In areas with significant silica exposure, there is an increased incidence in other autoimmune diseases such as systemic lupus erythematosus (SLE) with more male predominance [3]. In select animal models, exposure to silica and asbestos result in elevated ANA titers with IgG and C3 kidney deposits, simulating SLE-like disease. Following inhalation to the lungs, silica particles can mobilize to the kidney resulting in end stage renal disease in 5% of exposed individuals [3,4]. Our patient eventually developed nephrotic range proteinuria with renal biopsy confirming MCD and FSGS overlap. It is unclear what role the silica exposure played in the development of the kidney disease but his kidney biopsy did not reveal any immune deposits or silica crystals [5]. In a meta-analysis by Möhner et al, they highlighted the challenge in accurately characterizing the causal relationship between Silica and chronic non-malignant renal disease due to significant lag time between exposure and diagnosis [5]. Furthermore, it is difficult to identify a dose dependent relationship that could be attributed to an overall increase in mortality.

As an antigen, silica induces stimulation of bronchus associated lymphoid tissue (BALT), which is the mucosa associated lymphoid tissue (MALT) of the lung [3,6]. In normal healthy adults, BALT is either absent or if present lacks functional germinal centers. However, with chronic stimulation in the presence of an antigen, smoking or autoimmune diseases such as RA and SLE, it can mature with formation of the four germinal centers, triggering a malignant transformation [6]. Though silica is classified as a carcinogen by the International Agency for research on cancer (IARC), there is no documented association between silica and malignant lymphoma [4,7].

Following the diagnosis of Caplan’s syndrome, PFTs usually remain normal or have a mild obstructive physiology [1]. However, there have been case reports where patients have presented with a mixed restrictive and obstructive pattern depending on extent of the nodules, progressive parenchymal distortion and associated fibrosis [8]. Usually, the necrobiotic nodules remain peripheral and asymptomatic but they can also cavitate, enlarge and coalesce resulting in progressive massive fibrosis (PMF) [1]. In a retrospective case series by Constantinidis et al, they were able to demonstrate that patients with PMF had reduced vital capacity and significant airflow obstruction compared to patients with lesions that are localized to the periphery [9]. Our patients’ pulmonary status has remained the same over the course of 3 years.

In conclusion, Caplan’s syndrome remains a rare disease entity. Clear understanding of the exact causality between silica, RA and autoimmunity remains unclear. Diagnosis requires a thorough occupational health history that spans the patient’s adult life as well as second hand exposures during childhood. Differential diagnosis for these nodules includes lymphoma, tuberculosis, sarcoidosis or metastasis. Diagnostic sampling of the nodule is necessary not only to identify the necrobiotic nodules but to also rule out other similar disease processes. There is currently no evidence-based guideline on ways to routinely follow up these nodules and each case is addressed as an individual entity. However, patients should be monitored for rare complications such as hemoptysis or pneumothorax from the cavitated nodules as well as end stage pulmonary fibrosis.

## Learning Points/Take Home Messages

- Caplan’s syndrome is seen in patient with RA and chronic inorganic dust exposure
- Silica can trigger the immune system creating circulating autoantibodies and immune complexes
- Rare pulmonary complications include PMF, hemoptysis and pneumothorax from cavitated nodules
- Silica and auto antibodies can result in end stage renal disease
- There is no current guideline for silica exposure and cancer surveillance. It is recommended that patients follow age appropriate cancer screenings.
